# Molecular Diagnosis in Patients With Tuberous Sclerosis Complex: A Deep Sequencing Approach in Clinical Practice

**DOI:** 10.7759/cureus.110088

**Published:** 2026-06-02

**Authors:** Joana A Neto, Jacinta Fonseca, Cláudia Melo, Raquel Sousa, Marta Carvalho, Diogo F Rocha, João Parente Freixo, Miguel Leão, Ana Grangeia, Mafalda Sampaio

**Affiliations:** 1 Pediatric Neurology Unit, Department of Pediatrics, Hospital de São João, Porto, PRT; 2 Department of Neurology, Hospital de São João, Porto, PRT; 3 Neurogenetics Unit, Department of Medical Genetics, Hospital de São João, Porto, PRT; 4 Department of Genetics, Center for Predictive and Preventive Genetics, Institute of Molecular and Cell Biology, University of Porto, Porto, PRT

**Keywords:** genetic counseling, intronic variant, mosaiscism, next generation sequence analysis, tuberous sclerosis complex (tsc)

## Abstract

Background

Tuberous sclerosis complex (TSC) is an autosomal dominant neurocutaneous disorder caused by pathogenic variants in TSC1 or TSC2. Despite established clinical diagnostic criteria, a subset of patients remains molecularly unsolved after conventional genetic screening, often due to deep intronic variants, structural anomalies, or low-level somatic mosaicism.

Methods

A retrospective cohort study was performed. Patients without an identified pathogenic variant after conventional Sanger sequencing and multiplex ligation-dependent probe amplification (MLPA) were selected for targeted high-depth next-generation sequencing (NGS), achieving a median sequencing depth of at least 800×. Clinical data were collected for phenotypic characterization.

Results

High-depth targeted sequencing identified pathogenic or likely pathogenic variants in the TSC2 gene in four out of seven previously unresolved patients, resulting in an incremental diagnostic yield of 57.1% (4/7). The identified variants were deep intronic structural alterations or splice-modifying changes (c.2838-122G>A, c.848+281C>T, and c.2640-16_2640-8del). Two unrelated patients carried the identical c.848+281C>T deep intronic variant. Patients with identified TSC2 variants exhibited a high prevalence of structural central nervous system anomalies, epilepsy, and multi-system tumor involvement compared to the molecularly unconfirmed subgroup.

Discussion

High-depth targeted sequencing using hybrid-capture enrichment provides a substantial diagnostic advantage for detecting deep intronic variants in previously unsolved TSC cases. These findings advocate for integrating deep sequencing into clinical practice. Although TSC remains mainly a clinical diagnosis, identifying pathogenic variants has an important role in diagnosing patients who do not meet complete clinical criteria, in providing genetic counseling, as well as access to potential targeted therapies.

## Introduction

Tuberous sclerosis complex (TSC) (TSC1, 9q34: OMIM #191100; TSC2, 16p13.3: OMIM #613254) is an autosomal dominant neurocutaneous disorder with an estimated birth incidence of one in 6,000 to one in 10,000 live births, with an overall prevalence of one in 20,000 [1-3]. The condition exhibits an exceptionally broad phenotypic spectrum, featuring multi-system benign tumors (hamartomas) affecting organs such as the brain, skin, kidneys, heart, and lungs. These manifestations arise directly from hyperactivation of the mechanistic target of rapamycin complex 1 (mTORC1) pathway due to biallelic inactivation of either the TSC1 or TSC2 tumor suppressor genes [4,5]. Common clinical manifestations include neurodevelopmental impairment, autism spectrum disorder (ASD), and treatment-resistant epilepsy [4,5].

Historical diagnostic cohorts suggested that approximately 10% to 25% of individuals meeting definite clinical criteria for TSC lacked an identifiable pathogenic variant via conventional diagnostic screening, such as Sanger sequencing and exon-targeted deletion/duplication testing. Recent advances in next-generation sequencing (NGS), specifically ultra-deep targeted sequencing, have substantially reduced the proportion of these cases. The advanced platforms have uncovered previously hidden molecular etiologies, including deep intronic variants altering mRNA splicing, complex structural rearrangements, and low-level somatic or germline mosaicism. For instance, in the EPISTOP cohort [6], the utilization of highly sensitive, deep sequencing strategies identified pathogenic variants in all affected infants, revealing that somatic mosaicism accounted for approximately 10% of cases.

Somatic mosaicism in TSC represents a significant diagnostic challenge. Because these post-zygotic mutations occur after fertilization, the mutant allele is present in only a fraction of cells, often resulting in a variant allele fraction (VAF) well below the expected 50% baseline for heterozygous germline variants [6,7]. Furthermore, the detection rate is heavily dependent on the tissue source; mosaic variants may be present at lower, undetectable frequencies in peripheral blood-derived DNA compared to affected skin or hamartomatous tissue biopsies [6,7]. Despite the clinical utility of deep targeted panels, widespread access to these high-depth sequencing strategies remains restricted in routine clinical workflows due to regional logistical constraints and historical economic considerations.

In this study, we assessed the diagnostic yield of modern, high-depth targeted NGS in individuals with a definitive clinical diagnosis of TSC who lacked molecular confirmation after conventional screening. Furthermore, we aim to contextualize our findings within recently proposed genetic diagnostic algorithms [8] to optimize multi-tiered evaluation and facilitate earlier, precision-guided clinical management.

## Materials and methods

Study design and patient recruitment

A retrospective evaluation was performed on a cohort of patients clinically diagnosed with TSC and followed longitudinally at our tertiary referral center. From this baseline cohort, patients were selected for high-depth targeted sequencing based on the following inclusion criteria: (1) a definite clinical diagnosis of TSC established in accordance with the 2021 updated International TSC Consensus Group diagnostic criteria [2]; and (2) a previous negative molecular result for disease-causing variants after conventional screening via TSC1/TSC2 bidirectional Sanger sequencing and multiplex ligation-dependent probe amplification (MLPA). No explicit exclusion criteria were imposed. Unresolved patients were actively recruited between June 2023 and February 2024. All participants or their legal guardians provided written informed consent for peripheral blood collection and subsequent comprehensive genetic sequencing. This study was approved by the Institutional Ethics Committee of the Unidade Local de Saúde de São João (approval number CE-54-23, approved in February 2023).

Sequencing and data analysis

Following automated genomic DNA extraction from peripheral blood samples, DNA concentration and structural integrity were verified through standardized laboratory internal quality control criteria evaluated dynamically after each upstream processing step (including fragmentation and library preparation). NGS was performed externally at a College of American Pathologists (CAP)-accredited, Clinical Laboratory Improvement Amendments (CLIA)-certified molecular diagnostic laboratory (CeGaT GmbH, Tübingen, Germany). Target enrichment was executed using an in-solution, hybrid-capture probe technology designed to capture all protein-coding domains, flanking intronic segments (±8 bp), and disease-relevant non-coding areas (±30 bp) of the TSC1 and TSC2 loci. The enriched genomic libraries were sequenced using high-throughput paired-end sequencing on the Illumina NovaSeq 6000/NovaSeq X Plus platform (Illumina, Inc., San Diego, CA).

To ensure maximum sensitivity for low-frequency variants and intronic structural alterations, a strict post-sequencing vertical coverage threshold was enforced.

The diagnostic assay achieved an overall mean coverage depth of 800×, with 99.46% of all targeted nucleotides covered by at least 30 independent, high-quality reads per base. Any isolated targeted regions failing to meet these strict quality benchmarks were automatically rescued and cross-validated via classical bidirectional Sanger sequencing. Semi-quantitative assessment of copy number variations (CNVs) was computationally evaluated across all nuclear-encoded target loci by measuring normalized sample coverage profiles against an adjusted, multi-sample expected coverage baseline matrix optimized to eliminate inter-run microenvironmental variances.

Variants were classified and reported based on American College of Medical Genetics guidelines [9]. To substantiate the pathogenicity classifications of identified intronic and splice-site variants, multiple lines of evidence were integrated: population allele frequencies from the Genome Aggregation Database (gnomAD v4), peer-reviewed literature documentation, and consensus in silico outputs. SpliceAI scores > 0.2 were considered indicative of potential splicing alterations, with scores > 0.5 treated as high-probability splice-disrupting mutations.

Low-frequency somatic mosaicism was not explicitly assessed, as the computational pipeline was optimized specifically for germline variant detection, rendering the identification of low-level mosaic variants with an expected VAF < 20% was considered outside the validated limit of detection for this specific workflow. Computational analyses and in silico predictions were performed using the programs MetaLR [10] and SpliceAl [11].

Phenotype assessment

Demographic details and comprehensive clinical phenotypes were gathered via systematic electronic medical record reviews. Collected parameters included detailed documentation of neurological, neuropsychiatric, cutaneous, renal, cardiac, and pulmonary manifestations of TSC, alongside relevant imaging reports (brain MRI, renal and abdominal ultrasound/CT, and echocardiography).

## Results

Participants

From the cohort of 59 patients who met the 2021 diagnostic criteria for TSC [12] and underwent TSC1/TSC2 Sanger sequencing and MLPA, seven (12%) patients remained without genetic confirmation. 

Within this targeted study subset (n = 7), six patients (86%) were male, aged two to 30 years (median 14 years), with a median age at diagnosis of 2.25 years (inter-quartile range 0.56-5.25 years). The detailed clinical characteristics of these seven patients are provided in Table 1.

**Table 1:** Demographic and clinical data of TSC patients without molecular confirmation after Sanger sequencing and MLPA *: age at the clinical diagnosis of TSC; -: clinical feature not identified in patient; MLPA: multiplex ligation-dependent probe amplification; TSC: tuberous sclerosis complex; X: clinical feature identified in patient

Patient	1	2	3	4	5	6	7
Sex	M	M	M	M	M	M	F
Age, years	30	5	10	14	21	2	17
Age at diagnosis, years*	6	0.25	0	3	8	1.5	6
Family history TSC	-	X	-	-	-	-	-
Treatment with everolimus	X	X	-	-	-	-	X
Clinical features							
Central nervous system							
Epilepsy	X	X	X	X	-	-	-
Retinal hamartomas	X	-	X	-	-	-	-
Cortical dysplasias and subcortical tubers	X	-	-	X	X	-	X
Subependymal nodules	X	X	X	X	X	X	-
Subependymal giant cell astrocytoma	-	-	-	-	-	-	-
Cutaneous manifestations							
Hypopigmented macules	X	X	X	X	-	-	-
Facial angiofibromas	X	-	X	X	X	-	-
Ungual fibromas	-	-	X	-	-	-	-
Shagreen patch	-	X	-	-	-	-	-
Cardiac rhabdomyomas	X	X	X	-	-	X	-
Renal involvement							
Multiple renal cysts	X	-	X	-	-	-	-
Renal angiomyolipomas	X	-	-	-	X	-	X
Lymphangioleiomyomatosis	-	-	-	-	-	-	-
TSC-associated neuropsychiatric disorders							
Intellectual disability	X	X	X	-	-	-	-
Autism spectrum disorder	-	X	-	-	-	-	-

Regarding central nervous system (CNS) and neuropsychiatric disorders, all seven (100%) patients demonstrate brain magnetic resonance imaging (MRI) abnormalities, including subependymal nodules, cortical dysplasia, or subcortical tubers. Four patients (57%: Patients 1-4) had epilepsy. In this cohort, there were no subependymal giant cell astrocytoma (SEGA) identified during the study window. Comprehensive neuropsychiatric evaluations demonstrated intellectual disability in three patients (42.9%; Patients 1-3) and ASD in one patient (14.3%; patient 2).

Cutaneous manifestations included hypopigmented macules in four patients (57%: Patients 1-4) and facial angiofibromas also in four patients (57%: Patients 1, 3, 4, and 5), while shagreen patches were found in one patient (14%: Patient 2). There were no lymphangioleiomyomatosis identified.

Multi-system tumor involvement included cardiac rhabdomyomas, which were identified in four patients (57%: Patients 1, 2, 3, and 5). Renal involvement was characterized by multiple renal cysts in two patients (29%; Patients 1 and 3) and renal angiomyolipomas in three patients (43%: Patients 1, 5, and 7). Due to the progressive growth of renal AMLs, targeted medical therapy with the mTOR inhibitor everolimus was initiated in two patients (29%; Patients 1 and 5). Everolimus was also prescribed to one patient (14%; Patient 2) for the management of refractory, drug-resistant epilepsy.

Genetic variants and diagnostic yield

High-depth targeted sequencing successfully identified pathogenic or likely pathogenic variants within the TSC2 gene in four out of the seven previously unresolved patients, resulting in an incremental diagnostic yield of 57.1% within this selective NMI subgroup. No pathogenic variants were identified in the TSC1 gene. All detected variants were found in a constitutional, heterozygous state with standard germline VAFs clustering tightly between 44% and 53%. The molecular details and ACMG classification criteria for the identified variants are compiled in Table 2.

**Table 2:** Genotypic characterization of patients after deep sequencing *: not applicable; Pt: patient a: American College of Medical Genetics and Genomics (ACMG) Criteria: PVS1, PS4, PM2_Supporting, PS2 - Proven de novo literature; confirmed by functional RNA analysis causing a premature stop codon. SpliceAI Metric (Delta Score): 0.91. In Silico Splicing Consequence: Pseudoexon Activation. b: ACMG Criteria: PS3_Moderate, PM2_Supporting, PP3 - Recurrent deep intronic target; highly adverse in silico splice predictions; absent in controls. SpliceAI Metric (Delta Score): 0.72. In Silico Splicing Consequence: Cryptic Donor Activation. c: ACMG Criteria: PS3_Moderate, PM2_Supporting, PP3 - Same variant as Pt 2 (recurrent finding further reinforcing the aggregate clinical evidence). SpliceAI Metric (Delta Score): 0.72. In Silico Splicing Consequence: Cryptic Donor Activation. d: PVS1_Moderate, PM2_Supporting, PP3 - Deletion disrupts the necessary intronic polypyrimidine tract, heavily altering standard splice efficiency. SpliceAI Metric (Delta Score): 0.81. In Silico Splicing Consequence: Exon Skipping. Note: All variants listed are absent from the global population databases gnomAD v4. To the author’s knowledge, none of these patients is related.

Pt	Gene	Genomic position	cDNA	Reads	Variant classification	Genetic transmission
1	TSC2	c16:2127477G>A	c.2838-122G>A	891	Pathogenic	Heterozygosity
2	TSC2	c16:2107460C>T	c.848+281C>T	942	Likely pathogenic	Heterozygosity
3	TSC2	c16:2107460C>T	c.848+281C>T	1029	Likely pathogenic	Heterozygosity
4	TSC2	c16:2126051-2126060 GCCCCCTTCT>G	c.2640-16_2640-8del	905	Likely pathogenic	Heterozygosity
5	*	*	*	901	*	*
6	*	*	*	898	*	*
7	*	*	*	888	*	*

Two identical deep intronic variants, TSC2 c.848+281C>T, were identified in both Patients 2 and 3. Extended clinical histories confirmed that these two pediatric patients are entirely unrelated, residing in distinct geographical regions without known consanguinity or shared ancestry. 

Patient 4 presented a compelling, positive three-generation family history of TSC, with clinical manifestations documented in the patient’s brother, mother, maternal aunt, and maternal grandmother. Segregation testing confirmed that the TSC2 c.2640-16_2640-8del variant segregated with the disease phenotype across all accessible affected family members.

In contrast, Patients 5-7 remain molecularly unsolved, with no pathogenic sequence variants or structural copy number alterations detected across the TSC1 or TSC2 targeted regions.

Genotype-phenotype correlation

A descriptive comparison revealed that the four patients with identified TSC2 variants (Patients 1-4) exhibited clinically severe phenotypes characterized by a higher burden of neurological and multisystem manifestations. All four TSC2-positive patients presented with active epilepsy, and three required multi-drug anticonvulsant regimens. Neuropsychiatric comorbidities, including intellectual disability and ASD, were exclusively documented within this molecularly confirmed subgroup (Patients 1-3).

Cutaneous features were also prominent in the TSC2-positive group, with hypopigmented macules and facial angiofibromas identified in all four individuals, alongside the cohort's single instance of a shagreen patch (Patient 2). Structural multi-system tumor involvement, notably cardiac rhabdomyomas and complex renal cystic changes, was predominantly clustered within the TSC2-positive subset.

Conversely, the three patients who remained molecularly unconfirmed (Patients 5-7) presented with comparatively milder neurological phenotypes; none had experienced clinical seizures or demonstrated neuropsychiatric impairment up to the time of this report. Within this unsolved subgroup, clinical features were mostly restricted to isolated structural findings, such as late-onset facial angiofibromas (Patient 5), isolated cardiac rhabdomyomas (Patient 5), or isolated unilateral renal angiomyolipomas (Patients 5 and 7).

## Discussion

The application of high-depth targeted NGS resulted in an incremental diagnostic yield of 57% among patients with a definitive clinical diagnosis of TSC who had remained molecularly unsolved after conventional Sanger sequencing and MLPA screening. This finding demonstrates the diagnostic advantage of utilizing high-depth assays to re-evaluate NMI cohorts. The high yield achieved here underscores the presence of non-coding, splice-altering, and deep intronic variants that are systematically missed by classical diagnostic strategies but remain accessible via hybrid-capture high-depth approaches, directly aligning with findings from larger international cohorts [6,7].

A key finding from this study is the characterization of the deep intronic variants. The identification of TSC2 c.2838-122G>A and c.848+281C>T highlights the critical importance of evaluating non-coding variation. These variants are located deep within intronic boundaries, far outside the standard ±2 bp or ±8 bp windows typically analyzed by legacy diagnostic protocols.

The c.848+281C>T variant acts as a deleterious retention mutation by creating a high-scoring cryptic splice donor site that activates a cryptic 118 bp pseudoexon, introducing a premature stop codon into the mature mRNA transcript. Its independent detection as a de novo event in two completely unrelated patients in our small cohort highlights it as a potential non-coding mutational hotspot. The c.2838-122G>A variant similarly generates a novel de novo splice donor site, leading to aberrant intronic inclusion and subsequent frame-shift translation. The c.2640-16_2640-8del variant deletes a vital portion of the polypyrimidine tract directly upstream of the exon 22 splice acceptor site, inducing total exon skipping.

The pathogenic and likely pathogenic classifications of these non-coding variants are supported by strong consensus from advanced in silico neural network tools (SpliceAI scores of 0.72, 0.91, and 0.81, respectively) and are further validated by their complete absence from large global population databases such as gnomAD v4.

Our descriptive clinical observations - wherein all identified pathogenic variants were located within the TSC2 gene and coupled with severe multi-system and neurological manifestations - align with established literature indicating that TSC2 disruptions generally cause a more pronounced clinical phenotype than TSC1 alterations [4,6-8]. However, our cohort is subject to significant selection bias. As a specialized tertiary referral center, our clinic primarily manages patients with severe, drug-resistant epilepsy and advanced multi-system tumor burdens. Milder clinical phenotypes may be underrepresented in our longitudinal repository, which likely accounts for the exclusive detection of TSC2 mutations in our sequencing subset.

A key methodological limitation of this study involves the technical parameters of the bioinformatic pipeline utilized. Because the sequencing workflow was optimized specifically for high-fidelity germline variant identification, the variant-calling algorithms utilized an enforced interpretation and reporting threshold of >20% VAF. This design parameter effectively excludes the reliable identification of low-frequency somatic mosaicism. In tissue ecosystems with low mutant yields - such as peripheral blood samples - true mosaic variants frequently present with a VAF between 1% and 15%. At our vertical depth of 891 times, variations below 20% cannot be confidently distinguished from background sequencing noise or PCR fidelity limitations without a dedicated, mosaic-optimized bioinformatic pipeline. Consequently, we cannot exclude the possibility that the three remaining unsolved patients harbor underlying low-level somatic mosaicism.

To achieve definitive molecular confirmation in the remaining 43% (3/7) of our unresolved study subset, alternative diagnostic approaches should be considered (1) multi-tissue biopsies (namely, obtaining DNA from affected hamartomatous tissue which typically harbor a significantly higher concentration of the mutant allele) [13], (2) dedicated ultra-deep mosaic sequencing (re-sequencing blood or tissue DNA at ultra-high depths (5,000 times to 10,000 times) paired with specialized bioinformatic algorithms) [6], or (3) long-read sequencing and transcriptome analysis (using long-read sequencing technologies to capture complex, large-scale structural rearrangements or inversions, complemented by RNA sequencing).

While our study supports recently proposed genetic diagnostic frameworks [8] that advocate for the early integration of high-depth sequencing, we advise caution regarding the recommendation of deep sequencing as a universal, first-line diagnostic test. Implementing such high-depth, specialized assays as an initial screening tool may introduce notable logistical challenges and economic considerations for many healthcare systems. In resource-constrained settings, a tiered diagnostic approach - utilizing standard-depth NGS capture panels with built-in copy number variants (CNV) calling capability as the first tier, followed by reflexive high-depth sequencing or multi-tissue analysis for unresolved cases - remains a highly pragmatic and cost-effective strategy.

## Conclusions

In conclusion, our study underscores the utility of high-depth targeted NGS using hybrid-capture enrichment for resolving NMI cases of TSC. By expanding the analytical window to cover deep intronic regions and applying deep sequence depth, we successfully achieved an incremental diagnostic yield of 57% within a highly selected, previously unsolved cohort. The identification of independent, non-coding TSC2 variants emphasizes the need to look beyond the immediate exonic boundaries in classical NMI patients.

While germline-optimized sequencing pipelines are technically constrained by an interpretation threshold of >20% VAF, preventing the rule-out of low-frequency mosaicism from blood, their clinical implementation provides critical data to guide personalized medical therapies, family planning, and genetic counseling. Unresolved cases meeting definite clinical criteria should be reflexively routed toward advanced multi-tissue analyses, ultra-deep mosaic-validated sequencing, or RNA-based functional assays to optimize diagnostic sensitivity.
